# The EUPHRATES trial (Evaluating the Use of Polymyxin B Hemoperfusion in a Randomized controlled trial of Adults Treated for Endotoxemia and Septic shock): study protocol for a randomized controlled trial

**DOI:** 10.1186/1745-6215-15-218

**Published:** 2014-06-11

**Authors:** David J Klein, Debra Foster, Christa A Schorr, Kazem Kazempour, Paul M Walker, R Phillip Dellinger

**Affiliations:** 1 Department of Critical Care, and the Keenan Research Centre, Li Ka Shing Knowledge Institute, 4-054C Donnelly Wing, St Michael’s Hospital, 30 Bond Street, Toronto, Ontario M5B 1 W8, Canada; 2 Spectral Diagnostics Inc., -2 The West Mall, Toronto, Ontario M9C 1C2, Canada; 3 Cooper University Hospital, Cooper Medical School Rowan University, 1 Cooper Plaza, Camden, NJ 08103, USA; 4 Amarex Clinical Research, 20201 Century Blvd, 4th Floor, Germantown, MD 20874, USA

**Keywords:** Endotoxin, Endotoxemia, Septic shock, Polymyxin-B, Hemoperfusion, Theragnostics

## Abstract

**Background:**

Septic shock is common and has unacceptably high morbidity, mortality, and associated cost with numerous failed attempts at developing effective therapies. Endotoxin, one of the most potent mediators of sepsis, is found in high levels in approximately 50% of patients with septic shock. Polymyxin B (PMX) hemoperfusion has been shown in numerous studies to successfully remove endotoxin and potentially improve outcomes. EUPHRATES (Evaluating the Use of Polymyxin B Hemoperfusion in a Randomized controlled trial of Adults Treated for Endotoxemia and Septic shock) is a theragnostic trial (matching blood measurement to treatment capability) of PMX hemoperfusion in patients with septic shock and confirmed endotoxemia as measured by the endotoxin activity assay (EAA).

**Methods:**

EUPHRATES is a pivotal regulatory trial that is multi-centered, placebo-controlled and blinded. The trial is being conducted in fifty ICUs in the United States and Canada and is powered to enroll 360 patients. Patients with persistent septic shock despite adequate fluid resuscitation on vasopressors for more than 2 and less than 30 hours are eligible for measurement of the EAA. Those with EAA ≥0.60 are eligible to be randomized to treatment with two sessions of PMX hemoperfusion 24 hours apart. The primary endpoint for the trial is 28-day all-cause mortality.

**Discussion:**

Unique features of the trial include absence of systemic inflammatory response (SIRS) criteria as a requirement for inclusion, use of the EAA to confirm endotoxemia as a requisite for treatment, and use of a detailed “façade” hemoperfusion event as a blinding mechanism. The outcomes of the second interim analysis included a resizing of the trial to 650 patients and the addition of an exclusion criterion of subjects with multiple organ dysfunction score (MODS) ≤ 9. Results are anticipated in 2016.

**Trial registration:**

Clinicaltrials.gov identifier: NCT01046669. Registered: January 8, 2010.

## Background

Despite great advances in our understanding of the pathophysiology of severe sepsis (sepsis associated with organ dysfunction) and septic shock (severe sepsis associated with need for vasopressors following adequate fluid resuscitation), attempts to translate these findings into novel treatments and improved outcomes for patients have been disappointing [1]. Mortality, morbidity, and costs associated with management of septic shock patients in ICUs are unacceptably high [2]. Even the most promising of potential new therapies targeting specific mediators of the host response have failed to demonstrate consistent benefit when tested in large multi-centered, randomized, controlled trials despite encouraging results in animals and earlier phase studies [3]. Reasons cited for these failures are wide ranging, but likely include poor patient selection for therapies due to the heterogeneous and syndromic definition of the disease state of sepsis using American College of Chest Physicians / Society of Critical Care Medicine consensus criteria as well as failure of these therapies when subjected to the design rigors and mandated mortality outcomes found in late phase regulatory pathway trials [4].

Endotoxin (lipopolysaccharide; LPS) is perhaps the best studied and one of the most potent initiators and mediators of the host response in sepsis. Studies measuring endotoxin levels in septic patients have consistently demonstrated three key findings. First, a dose-response relationship between endotoxin levels in septic patients and adverse outcomes including organ failure and death [5]; second, the presence of elevated levels of circulating endotoxin in some, but not all, patients with sepsis including some with Gram positive infections and other forms of sepsis likely through the mechanism of gut translocation [5,6]; and third, the persistence of endotoxemia often days into the course of the disease process of severe sepsis [7]. A variety of approaches have been tried to target endotoxin therapeutically including monoclonal antibodies to neutralize LPS, small molecule and other competitive inhibitors of the LPS receptor Toll-like receptor (TLR)4 to block LPS signaling, use of specific molecules to enhance endotoxin deactivation and clearance, and extracorporeal devices to remove endotoxin [8]. In 1982, Ziegler and colleagues [9] first reported the successful treatment of a cohort of patients with Gram negative bacteremia and shock using a human anti-serum raised against the *Eschericia Coli* J5 bacterium core LPS or endotoxin. A 17% absolute reduction in death was observed in a selected group of 212 patients [9]. Of note, a very high percentage of these subjects had Gram negative bacteremia (63%), anticipated to correlate very highly with the presence of endotoxemia. Subsequently, two large projects both testing monoclonal antibodies targeting LPS in sepsis, HA-1A and E5, each demonstrated positive results in early phase studies [10,11] including a 1991 Ziegler and colleagues study [11] again demonstrating a 19% reduction in mortality in the subset of 200 patients with Gram negative bacteremia treated with HA-1A in a total sample of 543 patients. However, several large follow-up randomized controlled trials with both HA-1A as well as E5 were negative and neither drug was approved for clinical use in the United States [12,13]. More recently, additional attempts at neutralizing endotoxin to increase survival in severe sepsis utilizing (a) bactericidal/permeability-increasing protein, (b) a lipid emulsion of recombinant HDL that binds endotoxin, (c) a small molecule TLR4 inhibitor (TAK242), and (d) E5564, a lipid A analogue and competitive inhibitor of TLR4, also all failed to show reduction in mortality in severe sepsis patients [14-16].

Polymyxin B (PMX) is a cyclic cationic polypeptide antibiotic derived from *Bacillus polymyxa* that has the ability to bind and neutralize endotoxin. Unfortunately, infusion of polymyxin in humans results in nephrotoxicity and neurotoxicity limiting its intravenous use to salvage therapy for Gram negative enterobacteriaciae resistant to other antibiotics [17]. A novel therapeutic strategy whereby PMX is adsorbed to a polystyrene fiber in a hemoperfusion device that is used to remove circulating endotoxin was developed in Japan in the late 1990s. A wide variety of small open-label clinical trials using this strategy in sepsis and other patient subtypes have been published with generally encouraging results. Cruz and colleagues [18] published a systematic review and meta-analysis of 28 trials using PMX direct hemoperfusion (PMX-DHP) to treat patients with severe sepsis and septic shock. Although there was heterogeneity amongst the trials largely done in Europe and Japan, across a total cohort of 978 patients treated with the therapy, improvements were noted in hemodynamics (mean arterial pressure) as well as oxygenation (PaO2/FiO2 ratio, and there was a statistically significant improvement in risk of death (risk ratio 0.53, 95% CI 0.43 to 0.65) [18]. More recently, in 2009, the Early Use of Polymyxin Hemoperfusion in Abdominal Septic Shock (EUPHAS) trial [19], a randomized but unblinded study of 64 patients in 10 tertiary Italian ICUs, demonstrated statistically significant improvements in the primary endpoints of hemodynamics and organ dysfunction. Also, the absolute risk of death at 28 days improved significantly from 53% in the conventional therapy group to 32% in the PMX-DHP treated group [19]. This result, albeit encouraging, is considered controversial as the trial was stopped early after an interim analysis showed the mortality difference in a secondary endpoint. In addition, the trial was not blinded [20]. Patients were selected for therapy based on evidence of septic shock from an intra-abdominal source to hopefully enrich the patient population, but endotoxin levels were not measured prior to enrollment - a common theme in all the previously discussed trials attempting to neutralize endotoxin with drug therapy. Thus, there have been widespread calls for a definitive randomized controlled trial on this promising but not widely accepted therapy in North America [20].

Here, we present the protocol for, and discuss the design, of the “Evaluating the Use of Polymyxin B Hemoperfusion in a Randomized controlled trial of Adults Treated for Endotoxemia and Septic shock (EUPHRATES)” trial. We believe it to be the first blinded and randomized controlled, diagnostic-directed trial of a hemoperfusion device ever done in sepsis. We hypothesize that targeted treatment of patients with septic shock and high levels of circulating endotoxin as confirmed by the endotoxin activity assay (EAA) combined with directed therapy in the form of PMX-DHP endotoxin removal, will result in a significant reduction in 28-day all-cause risk of death.

## Methods and design

### Study design and outcomes

**Figure 1:**
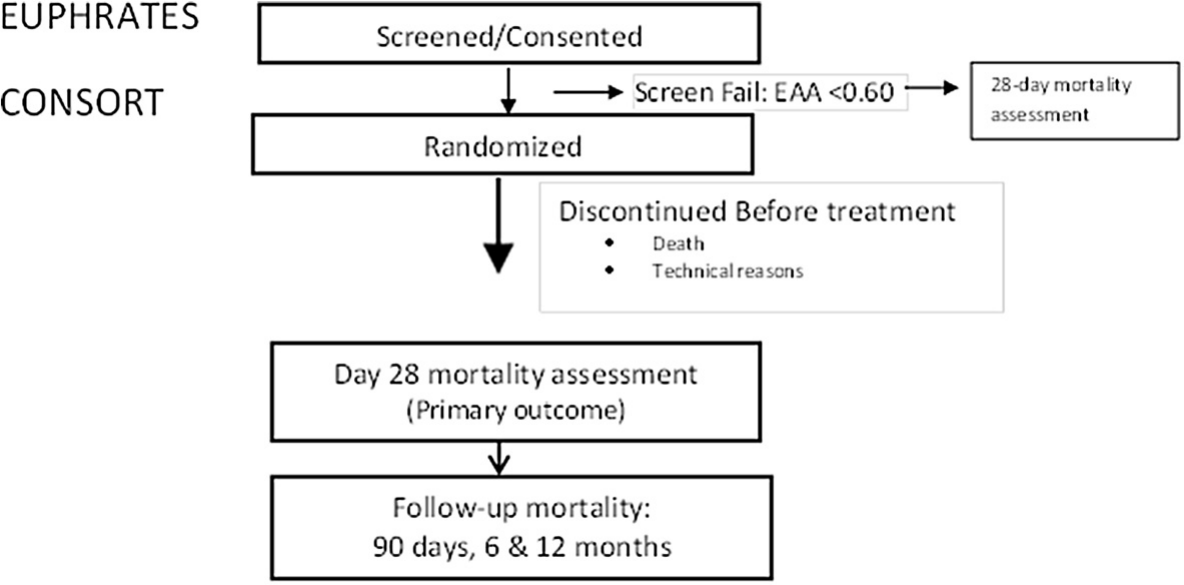
**Consort diagram for EUPHRATES (Evaluating the Use of Polymyxin B Hemoperfusion in a Randomized controlled trial of Adults Treated for Endotoxemia and Septic shock trial).** EAA, endotoxin activity assay.

The ongoing EUPHRATES trial (Clinicaltrials.gov identifier: NCT01046669) is a North American pivotal (under Investigational Device Exemption (IDE) from the United States Food and Drug Administration (FDA)) double-blinded theragnostic trial of PMX-DHP therapy in adult patients with septic shock and confirmed endotoxemia as measured by EAA. The trial began in June 2010 and is scheduled to be completed by the end of 2016. Ethics approval for the trial protocol has been obtained from the research ethics board responsible for each site prior to starting the trial. A list of all ethical bodies that reviewed and approved the trial is listed in Table 1. The primary outcome of the trial is 28-day all-cause mortality. The total number of patients estimated to be included in the study was planned as 360 from 50 ICUs in the United States and Canada. Consecutive patients with evidence of septic shock as indicated by initiation of intravenous antibiotics and on vasopressor therapy for hypotension will be screened and those eligible will be consented for a blood draw for measurement of EAA. The consort diagram is shown in Figure 1. Those with an EAA ≥0.60 (high endotoxin activity level) will be considered eligible and consented for randomization to potential treatment with PMX-DHP or the control group. Otherwise, eligible patients with an EAA <0.60 after up to two blood draws 4 to 6 hours apart will be not be randomized but will be followed using a minimal dataset for mortality at 28 days in an observational cohort study. It is estimated that approximately 50% of otherwise eligible patients will have an EAA ≥0.60. Table 2 summarizes the inclusion and exclusion criteria for EUPHRATES. Note that a patient with any type of infection producing septic shock is eligible to be enrolled if high endotoxin activity is present (EAA ≥0.60) as endotoxemia is not limited to Gram negative infection only (likely gut-derived endotoxin from translocation in non-Gram negative bacterial shock). In addition to the primary outcome measure of 28-day all-cause mortality, secondary outcomes include 90-day, 6-month and 1-year all-cause mortality. Hemodynamic outcomes will be followed using the cumulative vasopressor index (Table 3) and other organ failure outcomes will be captured as both total and component scores of the multiple organ dysfunction score (MODS) [21,22]. Renal outcomes will be tracked using the Acute Kidney Injury (AKIN) criteria [23]. A full list of secondary and exploratory objectives is listed in Table 4.

**Table 1:** Institutional Review Board information for EUPHRATES

**SDI-PMX-NA001 (EUPHRATES) site IRB list**			
**Site#**	**IRB Chair**	**IRB address**	**Approval date**
**Site #01**	Louis Zeiger, MD	Cooper Health System	18 Mar 2010
		Institutional Review Board	
		Three Cooper Plaza Suite 504 Camden, NJ 08103	
**Site #02**	Theodore D Schultz	Western Institutional Review Board	12 Jul 2010
		3535 Seventh Ave. SW	
		Olympia, WA 98502	
**Site #04 *(site closed)***	James Linakis	Lifespan Office Research Administration Committee on the Protection of Human Subjects	17 Sep 2010
		Rhode Island Hospital Institutional Review Board	
		593 Eddy St – Aldrich, 5 Providence, Rhode Island 02903	
**Site #05**	William Tremaine	Institutional Review Board	04 Mar 2011
		Office for Human Research Protection	
		201 Building, Room 4–60	
		200 First Street S.W.	
		Rochester, Minnesota 55905	
**Site #06**	Anne Dougherty, MD	The Committee for the Protection of Human Subjects	21 Dec 2010
		6410 Fannin St., Suite 1100	
		Houston, TX 77030	
**Site #07**	Donald York, PhD	Institutional Review Board	16 Aug 2010
		621 S. New Ballas, Suite 6002	
		St. Louis, Mo. 63141	
**Site #08**	Theodore D Schultz	Western Institutional Review Board	29 Nov 2010
		3535 Seventh Ave. SW	
		Olympia, WA 98502	
**Site #09**	Roger S Wilson, MD	Institutional Review Board/Privacy Board (IRB/PB)	14 Dec 2010
		Memorial Sloan Kettering Cancer Center	
		1275 York Avenue	
		New York, NY 10065	
**Site #10**	Ernest Prentice, PhD	Office of Regulatory Affairs	13 Dec 2010
		Institutional Review Board	
		987830 Nebraska Medical Center	
		Omaha, NE 68198-7830	
**Site #11**	Peter Lichtenthal, MD	Human Subjects Protection Program	22 Nov 2010
		1618 E. Helen Street	
		The University of Arizona	
		PO Box 245137	
		Tucson, AZ 85724	
**Site #12**	Glenn Markenson,MD and Rick Granowitz, MD	Baystate Medical Center	15 Dec 2010
		Institutional Review Board	
		759 Chestnut Street	
		Springfield, MA 01199	
**Site #13**	Theodore D Schultz	Western Institutional Review Board	24 Nov 2010
		3535 Seventh Ave. SW	
		Olympia, WA 98502	
**Site #16**	Theodore D Schultz	Western Institutional Review Board	04 April 2011
		3535 Seventh Ave. SW	
		Olympia, WA 98502	
**Site #17 *(site closed)***	Theodore D Schultz	Western Institutional Review Board	11 Apr 2011
		3535 Seventh Ave. SW	
		Olympia, WA 98502	
**Site #19**	Craig Scoville, MD, PhD	Eastern Idaho Regional Medical Center IRB	14 Mar 2011
		3100 Channing Way	
		Idaho Falls, ID 83404	
**Site #20a and 20b**	Mike Caligiuri	University of California San Diego	27 Oct 2011
		9500 Gilman Drive	
		La Jolla, California 92093	
**Site #21**	Jennifer Weinman, BA, MA	Washington University in St. Louis	21Feb 2012
		Human Research Protection Office	
		660 South Euclid Avenue	
		Campus Box 8089	
		St. Louis, MO 63110	
**Site #22**	J Bruce Smith, MD, CIP	Thomas Jefferson University Hospital	20 Oct 2011
		111 S. 11th Street	
		Philadelphia, PA 19107	
**Site #23a**	Theodore D Schultz	Western Institutional Review Board	29 Sep 2011
		3535 Seventh Ave. SW	
		Olympia, WA 98502	
**Site #23b**	Manoo Bhakta, MD	College of Medicine, Chattanooga	5 Jul 2012
		Scientific Review Board	
		960 East Third Street	
		Suite 102	
		Chattanooga, TN 37403	
**Site #25 *(site closed)***	John T Promes, MD	Orlando Health	20 Jan 2012
		1414 Kuhl Avenue	
		Orlando, FL 32806	
**Site #26**	SKM Kimber, MD, FRCPC	Health Research Ethics Board	9 Jan 2012
		308 Campus Towers	
		University of Alberta	
		Edmonton, Alberta, Canada T6G 1 KB	
**Site #27 *(site closed)***	Pamela J Oatis, MD	Research Oversight and Education	29 Feb 2012
		Mercy St. Vincent Medical Center	
		2213 Cherry Street	
		Toledo, OH 43608	
**Site #28 *(site closed)***	Lois Colliler	Sharp Corondado	18 Apr 2012
		Sharp Memorial Hospital	
		5555 Gossmont Center	
		La Mesa, CA 91942	
**Site #29a**	Russell Bjork, MD	Memorial Health System	27 Mar 2012
		1400 East Boulder	
		Colorado Springs, CO 80909	
**Site #29b**	Russell Bjork, MD	Memorial Health System	26 Oct 2012
		1400 East Boulder	
		Colorado Springs, CO 80909	
**Site #30**	Philip C Hébert, MD PhD FCFPC	Research Ethics Office, Room C819	11 Sep 2012
		2075 Bayview Avenue	
		Toronto, ON Canada M4N 3 M5	
**Site #31**	JD Miller, MD	System Center-Hazard	11 Apr 2012
		100 Airport Gardens Road	
		Hazard, KY 41701	
**Site #32**	Steven Kushner, MD	Helen F. Graham Cancer Center & Research Institute	01 Jul 2013
		West Pavilion - Suite 2350	
		4701 Ogletown-Stanton Road	
		Newark, Delaware 19713	
**Site #33**	John Hafner Jr, MD	Institutional Review Board	27 Jun 2012
		One Illini Drive	
		Box 1649	
		Peoria, IL 61656-1649	
**Sites #34a and #34b**	Vivek Singh, MD	Institutional Review Board Office	09 Aug 2012
		Mail Code 7830	
		7703 Floyd Curl Dr.	
		San Antonio, TX 78229-3900	
**Sites #35a and #35b**	Raphael Saginur, MD	Ottawa Hospital Research Ethics Board	30 Aug 2012
		725 Parkdale Ave.	
		Civic Box 411	
		LOEB Building	
		Ottawa, ON K1Y 4E9	
**Site #36**	Allen Korenbilt, MD, CIP	Rush University Medical Center	31 Oct 2012
		1653 West Congress Parkway	
		Chicago, IL 60612-3833	
**Site #37**	Theodore D Schultz	Western Institutional Review Board	17 Sep 2012
		3535 Seventh Ave. SW	
		Olympia, WA 98502	
**Site #38**	Theodore D Schultz	Western Institutional Review Board	24 Jan 2013
		3535 Seventh Ave. SW	
		Olympia, WA 98502	
**Site #39**	Leonardo Tamariz, MD, MPH	Human Studies Subcommittee	19 Sep 2012
		Miami VA Healthcare System	
		1201 Northwest 16th St.	
		Miami, FL 33125-1693	
**Site #40**	Theodore D Schultz	Western Institutional Review Board	17 Jan 2013
		3535 Seventh Ave. SW	
		Olympia, WA 98502	
**Site #42**	Jeffrey Silverstein, MD, CIP;	Icahn School of Medicine at Mount Sinai	01 Feb 2013
	Glenn Martin, MD, CIP;	One Gustave L. Levy Place	
	Ilene Wilets, PhD, CIP	Box 1081	
		New York, NY 10029-6574	
**Site #43**	John Montogomery, Chair	Lakeridge Health	04 Apr 2013
		1 Hospital Ct.	
		Oshawa, ON, Canada L1G 2B9	
**Site #44**	SKM Kimber, MD, FRCPC	308 Campus Tower	27 Feb 2013
		University of Alberta	
		Edmonton, AB T6G 1 K8	
**Site #45**	Franck Molin, MD	IUCPQ	15 Apr 2013
		2725, Chemin Sainte-Foy	
		Quebec, QC G1V 4G5	
**Site #46**	Alan Lichtin	Cleveland Clinic Foundation	05 Mar 2013
		9500 Euclid Ave.	
		Cleveland, OH 44195	
**Site #48**	Timothy Roehrs, PhD	Henry Ford Health System Research Administration	01 Apr2013
		2799 West Grand Blvd	
		CFP-bsmt, room 46	
		Detroit, MI 48202	
**Site #49**	Stacey A. Page, PhD, Chair, CHREB	Conjoint Health Research Ethics Board (CHREB)	10 Sep 2013
		Research Services Office	
		3rd Floor Mackimmie Library Tower (MLT 300)	
		2500 University Dr., NW	
		Calgary, AB, Canada T2N 1 N4	
**Site #51**	Ronald Heslegrave, PhD	Mount Sinai Hospital	03 Jul 2013
		Research Ethics Board	
		600 University Ave.	
		Room 19–311	
		Toronto, ON, Canada M5G 1X5	
**Site #52a and #52b**	Karen McRae, MD, Co-Chair/Multidisciplinary UHN REB	University Health Network (UHN)	15 Apr 2013
		Research Ethics Board (REB)	
		10th Floor, Room 1056	
		700 University Ave.	
		Toronto, ON, Canada M5G 1Z5	
**Site #53**	R Bert Wilkins	Western Institutional Review Board	22 Jul 2013
		3535 Seventh Ave. SW	
		Olympia, WA 98502	
**Site #54**	L Wiley Nifong, MD	UMCIRB	12 Feb 2014
		East Carolina University	
		Brody School of Medicine, Brody 4 N-70	
		Greenville, NC 27834	
**Site #55**	Rhodes L Rigsby, MD	Institutional Review Board	05 Sep 2013
		Research Protection Programs	
		24887 Taylor St.	
		Suite 202	
		Loma Linda, CA 92350	
**Site #56**	David Spiegel, MD	Stanford University Research	12 Jun 2012
		Compliance Office	
		1501 South California Ave.	
		MC: 5579	
		Palo Alto, CA 94304	
**Site #57**	Ike Eriator, MD	University of Mississippi Medical Center	12 Feb 2013
		Institutional Review Board	
		2500 North State St.	
		Jackson, MS 39216-4505	
**Site #58**	Michael Geisser, PhD, Co-Chair	University of Michigan, IRBMED	7 Mar 2014
		2800 Plymouth Road	
		Building 520, Room 3214	
		Ann Arbor, MI 48109-2800	

**Table 2:** Summary of inclusion and exclusion criteria

**Inclusion criteria**	**Exclusion criteria**
Subjects who meet the following criteria (and have a signed informed consent) will be allowed into the study:	1. Inability to obtain an informed consent from the subject, family member or an authorized surrogate
1. Age ≥18 years of age	2. Lack of commitment for full medical support
2. Hypotension requiring vasopressor support: Requirement for at least one of the vasopressors listed below, at the dose shown below, for at least 2 continuous hours and no more than 30 hours	3. Inability to achieve or maintain a minimum mean arterial pressure of ≥65 mmHg despite vasopressor therapy and fluid resuscitation
a. Norepinephrine >0.05 μg/kg/minute	4. Subject has end-stage renal disease and requires chronic dialysis
b. Dopamine >10 μg/kg/minute	5. There is clinical support for non-septic shock such as
c. Phenylephrine >0.4 μg/kg/minute	a. Acute pulmonary embolus
d. Epinephrine >0.05 μg/kg/minute	b. Transfusion reaction
e. Vasopressin >0.03 units/minute	c. Severe congestive heart failure (for example, New York Heart Association Class IV, ejection fraction <35%)
f. Vasopressin (any dose) in combination with another vasopressor listed above	6. Subject has had chest compressions as part of cardiopulmonary resuscitation this hospitalization without immediate return to communicative state
3. The subject must have received intravenous fluid resuscitation of a minimum 30 mL/kg administered within 24 hours of eligibility	
4. Documented or suspected infection defined as definitive or empiric intravenous antibiotic administration	7. Subject has had an acute myocardial infarction within the past 4 weeks
5. Endotoxin activity assay ≥0.60	8. Subject has uncontrolled hemorrhage (acute blood loss requiring >3 Units of Packed red blood cellsin the past 24 hours)
6. Evidence of at least one of the following criteria for new onset organ dysfunction that is considered to be due to the acute illness	9. Major trauma within 36 hours of screening
a. Requirement for positive pressure ventilation via an endotracheal tube or tracheostomy tube	10. Subject has severe granulocytopenia (leukocyte count <500 cells/mm^3^) or severe thrombocytopenia (platelet count <30,000 cells/mm^3^)
b. Thrombocytopenia defined as acute onset of platelet count <150,000 μ/L or a reduction of 50% from prior known levels	11. HIV infection in association with a last known or suspected CD4 count of <50/mm^3^
c. Acute oliguria defined as urine output <0.5 ml/kg/hour for at least 6 hours despite adequate fluid resuscitation	12. Subject’s baseline state is non-communicative
	13. Subject has sustained extensive third-degree burns within the past 7 days
	14. Body weight <35 kg
	15. Known hypersensitivity to polymyxin B
	16. Subject has known sensitivity or allergy to heparin or has a history of heparin associated thrombocytopenia
	17. Subject is currently enrolled in an investigational drug or device trial
	18. Subject has been previously enrolled in the current trial
	19. Any other condition, that in the opinion of the investigator, would preclude the subject from being a suitable candidate for enrolment, such as end stage chronic illness with no reasonable expectation of survival to hospital discharge
	20. Multiple Organ Dysfunction Score ≤9 ** - *added post-second interim analysis*

**Table 3:** Cumulative vasopressor index

**Vasopressor**	**Dose Range**	**Dose Range**	**Dose Range**	**Dose Range**
	**1 point**	**2 Points**	**3 Points**	**4 Points**
Dopamine	0 dose ≤ 5	5 < dose ≤ 10	10 < dose ≤ 15	> 15
(mcg/kg/min)				
Epinephrine	-	0 < dose < 0.05	0.05 < dose < 0.1	> 0.1
(mcg/kg/min)				
Norepinephrine	-	0 < dose < 0.05	0.05 < dose < 0.1	> 0.1
(mcg/kg/min)				
Phenylephrine	-	0 < dose < 0.4	0.4 < dose < 0.8	> 0.8
(mcg/kg/min)				
Vasopressin	-	-	-	Any dose
(units/min)				

**Table 4:** Secondary and exploratory outcomes

1.	To compare mortality between the two groups at 90 days, 6 months and 12 months post-start of treatment
2.	To compare the change in endotoxin levels between the PMX cartridge-treated group and the control group at 12 hours after completion of a second PMX cartridge, with a treatment target of ≥15% reduction of EAA levels with PMX cartridge treatment
3.	To compare the changes in vasopressor doses for the two groups from day 0 to day 3
4.	To compare the number of days of need for vasopressors in each group from day 0 to day 28 (days alive and off vasopressors)
5.	To compare changes in mean arterial blood pressure for the two groups from day 0 to day 3
6.	Comparison of the changes in renal function from day 0 to day 3:
i.	Fluid balance including urine output
ii.	Serum creatinine
7.	To compare the effects of two uses of the PMX cartridge on progression of, and recovery from, organ dysfunction using the multiple organ dysfunction score from day 0 to day 3
8.	To compare the number of days of need for renal replacement therapy in each group from day 0 to day 28 (days alive and off renal replacement therapy)
9.	To compare the number of days of need for mechanical ventilation in each group from day 0 to day 28 (days alive and off mechanical ventilation)
10.	To compare the mean number of days spent in hospital by subjects in each group for survivors to day 28
11.	To compare survival time from baseline to death within 28 days and compare the risk of death between the two study arms
12.	To compare survival time from baseline to death within 90 days and compare the risk of death between the two study arms

### Participants

Participants aged ≥18 years old with septic shock and high endotoxin activity (EAA ≥0.60) are eligible for the study. Written informed consent is obtained from each patient or their substitute decision maker prior to randomization. Septic shock is defined as a documented or suspected infection as indicated by either directed or empiric use of antibiotics and presence of hypotension of at least 2 hours duration requiring ongoing vasopressor support despite a fluid challenge of 30 ml/kg in the previous 24 hours. Subjects must also have at least one additional organ failure as defined by oliguria, thrombocytopenia, or need for mechanical ventilation. There is no requirement for subjects to meet any systemic inflammatory response syndrome criteria. Subjects with end-stage renal disease on dialysis are not eligible. The estimated 28-day mortality in this cohort of patients as per the Surviving Sepsis Campaign (SSC) database estimate is 35% [24].

Investigators agree to managing patients in line with the SSC guidelines [25,26]. There are no specific management requirements; however, as indicators of overall adherence to SSC guidelines and good clinical practices, two quality measures are being monitored.

### Study procedures

#### Coordination and randomization

A clinical coordinating center (C4) at Cooper University Hospital, Camden, NJ, USA, has been established to confirm subject eligibility for the study and provide support to investigators and study personnel throughout the study period. The C4 consists of four members to evaluate eligibility (sepsis members) and two nephrologists (nephrology members) who provide treatment-related support. Subjects approved for randomization based on meeting full eligibility criteria including an EAA ≥0.60 will be randomized. Randomization will be 1:1 using random block sizes of two and four. Research pharmacists will dispense identical boxes containing either treatment cartridge boxes or sham boxes based on a serial number code linked to the randomization scheme. The sepsis C4 members are blinded to treatment allocation while the nephrology C4 team can be unblinded as needed.

#### Timing

**Figure 2:**
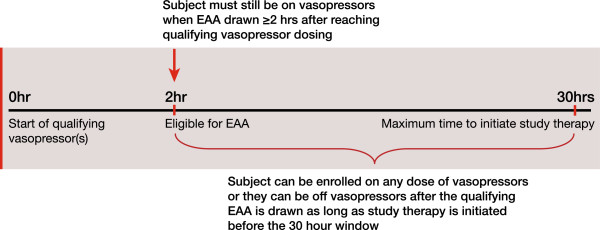
**Timing for patient identification and enrollment.** EAA, endotoxin activity assay.

**Figure 3:**
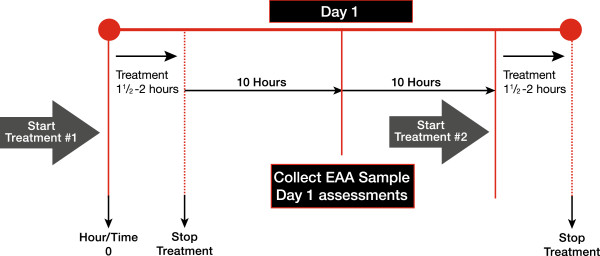
**Timeline for initiation of intervention.** EAA, endotoxin activity assay.

To be eligible for treatment, following a minimum fluid challenge, patients must be in septic shock and on vasopressors at the specified dose levels in Table 2 for at least 2 hours continuously and not for more than 30 hours. A sample timeline for consent, measurement of EAA, and randomization are shown in Figure 2. The first treatment must be initiated within 24 hours of the EAA consent and the second approximately 22 hours after the first treatment. A sample timeline for the intervention period is shown in Figure 3.

#### Measurement of endotoxin activity assay

The EAA received 510(k) *de novo* clearance from the FDA in 2003 for the measurement of clinical samples for endotoxemia and to assess patients for risk of severe sepsis. The assay is semi-quantitative and values are expressed as EAA units between 0 and 1. A low EAA level is considered to be <0.40, intermediate level 0.40 to 0.59, and high level ≥0.60 (Spectral Diagnostics Inc., Toronto, Ontario, Canada). These cutoffs were established based on the results of the MEDIC trial [5]. The details of how to perform the assay are published elsewhere [27]. In the current trial, samples are run in duplicate by trained personnel. A correlation value of 15% or better is required for EAA results less than <0.20, and 20% for all other EAA results. If a patient is otherwise eligible but the EAA is below the high threshold of 0.60, a follow-up test may be done once, 4 to 6 hours after the first test, to reassess for potential eligibility.

#### Description of the study intervention

Patients assigned to the treatment arm who do not already have access for dialysis will have a standard hemodialysis catheter inserted in one of the femoral veins by an unblinded physician not involved in the clinical care of the patient. For those patients not assigned to the treatment arm, a “cut” hemodialysis catheter and associated dressing will be affixed to the skin in the femoral position to simulate a newly inserted line. The same physician that would have inserted the catheter stays in the room behind a curtain for a similar amount of time as would have been predicted for catheter insertion. At the conclusion of each hemoperfusion session, a standard opaque dressing will be placed over the line site to maintain the blind. After the second hemoperfusion session, the hemodialysis catheter is removed and a standard opaque dressing applied over the region. In some patients the decision for dialysis has been made contemporaneously with the consent for study and in that case a dialysis catheter will be inserted and the treatment blinded only.

The PMX hemoperfusion column used in the study was developed in Japan and is manufactured by Toray Industries (Tokyo, Japan). The PMX treatment will be administered twice using two cartridges with the treatments completed approximately 24 hours apart. Each treatment will last for approximately 2 hours, at a blood flow rate of approximately 100 ml/minute through the circuit (range of 80 to 120 ml/minute). For the current trial, a standard intermittent hemodialysis machine will be used as a blood pump to administer the treatment. At the discretion of the unblinded nephrologist on the hemoperfusion team, anticoagulation, using intravenous unfractionated heparin, will be administered to maintain patency of the circuit for the duration of each 2-hour treatment. Patients already on anticoagulation for other indications or those with coagulopathy will not be anticoagulated further at the discretion of the nephrologist on the hemoperfusion team. Citrate anticoagulation is not allowed as per the recommendations of the manufacturer.

#### Blinding

The trial is blinded. For those patients assigned to the control arm, a simulated or “sham” hemoperfusion event is carried out to coincide with the timing of the treatment events in the other arm. In addition, a “cut” line is placed on the skin to simulate the presence of an indwelling central line. To maintain the trial’s blind, the ICU physician investigators, those health-care professionals involved in recording blinded data, and those who are involved in data analysis, will remain blinded to allocation of treatment.

A nephrology staff member, the study coordinator, the ICU bedside nurse, and a pharmacist will know the treatment allocation and are trained to record allocation and treatment records (timing of device use) and concomitant anti-coagulant medication administered (for example, heparin) on the source documents and case report forms (CRFs) that will be kept blinded from the remaining study personnel.

Nephrology staff will be trained to use the PMX cartridge on those subjects randomized to the treatment arm and to maintain the blind for the subjects that are randomized to the control arm by performing the “sham” perfusion event. Study staff (principal investigator and other ICU personnel involved in the subjects care) and the subject (and/or the subjects surrogate) will remain blinded to the study allocation. Pre-treatment EAA results will be made available to the treating physician, but all subsequent EAA results are blinded. During the period of time of the treatment intervention or sham perfusion event sites employ a number of methods to ensure the blind is maintained. During the treatment phase, a façade perfusion event is conducted whereby a dialysis instrument is brought to the patient’s bedside and curtains are drawn. A sign is attached to the curtain indicating that a study procedure is in progress. The unblinded nephrology team and/or study coordinator perform the hemoperfusion or sham perfusion for approximately 2 hours. This process is repeated once at approximately 22 hours such that the treatment of PMX or sham treatment is completed twice within a 24-hour period.

Breaks in the blind are reported to the sponsor via the medical monitor and are recorded in CRFs. Emergency unblinding procedures include confirmatory correspondence with the pharmacy of the true treatment allocation.

#### Sample size and power

The power analysis is based on a two-sided Fisher’s exact test to compare the proportions of death within or at 28 days between the PMX treatment and control groups. It is assumed that for the control arm of standard medical care alone approximately 35% subjects will die by day 28 after the enrollment. It is also hypothesized that there will be an approximately 15% improvement in 28-day mortality rate for subjects randomized who receive the standard medical care plus the PMX cartridge, as compared to those receiving the standard medical care alone. The overall significance level for the entire sequence of primary efficacy tests is assumed at 5% in a two-sided test. An interim analysis based upon safety only was conducted after 76 patients were enrolled in January 2013. No efficacy analysis was conducted. The data safety monitoring board (DSMB) noted no specific safety concerns at that time. There is one planned additional interim analysis approximately at the midpoint (that is, 180 subjects enrolled). Because of the planned interim analysis, the maximum sample size for the trial (that is, if the trial is not terminated by the interim analysis) is based on the final efficacy test to compare day 28 mortality rate between the two study arms at a significance level of 0.048 based on the method of O’Brien and Fleming [28].

Assuming a sample size ratio of 1:1 between the two study arms, a sample of 180 subjects per study arm (that is, a total of 360 subjects for both arms) provides at least 80% statistical power to detect the hypothesized effect size (that is, 15% difference) from the final primary efficacy test. The statistical power with the proposed sample size will thus be more than 80% from the entire sequence of the planned primary efficacy analyses. These power analyses assume a 15% attrition rate from the randomized subjects of each study arm. Therefore, a minimum of 306 evaluable subjects are necessary to achieve at least 80% statistical power based on the hypothesized effect size.

#### Monitoring

The trial is fully monitored by the contract research organization responsible for trial operations. A trial monitoring plan has been established and is on file with the FDA. One hundred percent source data verification is done for all data points in the CRFs. Protocol violations are predefined. Adherence to treatment as defined by number of treatments delivered versus prescribed as well as total time of each delivered treatment is compiled in the CRF and tabulated for analysis. All co-interventions are captured including concurrent medications. A detailed policy on co-enrollment in other studies has also been developed and approved by the DSMB.

#### Interim analysis

An interim analysis will be performed by an unblinded statistician independent of the DSMB and the Sponsor after the first 180 subjects (that is, about 90 per study arm, or about 50% of the total planned evaluable subjects) complete their day 28 assessment. The interim analysis will only analyze the portion of the data through day 28 for subjects who have completed the day 28 assessment and is based on the final efficacy test to compare day 28 mortality rate between the two study arms at a significance level of 0.048.

The primary purpose of the interim analysis is to determine if the trial should be terminated early or the study protocol should be modified. The primary population for this interim analysis will be the intention to treat (ITT) population. Secondary analyses will examine the modified ITT population - that is, all patients who have at least one treatment application. The issues to be addressed by the interim analysis will include the possibility that: (a) the treatment is clearly effective so that it is unnecessary to continue to the planned termination point insofar as the primary efficacy endpoint is concerned, *P* value is less than 0.005; or (b) the treatment is so ineffective that continuation of the study would provide little or no chance of ever reaching statistical significance. In addition to the efficacy assessment, at the interim analysis the DSMB will review all serious adverse events and unanticipated serious device effects to evaluate if there are medically significant justifications due to excessive side effects or safety concerns to demand an early end to the trial or a modification of the intervention. The interim analysis will thus evaluate both safety and efficacy variables.

If the trial is not terminated based on the interim analysis, the results of the interim efficacy analysis may be used to revise sample size estimations, which will serve in planning the remaining part of the trial. An independent statistician will conduct the power analyses and sample size re-estimation.

If the trial is terminated as a result of the interim analysis (that is, *P* value < 0.005) or by the recommendations of the DSMB, the final efficacy and safety analyses will be performed using the entire data of follow-up to 12 months or until the last assessment for all subjects randomized prior to the study termination date.

### Statistical analysis of results

Primary efficacy analyses will be conducted on the ITT population. The primary efficacy endpoint is the all-cause mortality rate of the subjects within 28 days after the initiation of the treatment perfusion or the sham perfusion. Let *P*_0_ and *P*_1_ be the proportion of death within 28 days for the arm of standard medical care alone and the arm of standard medical care plus the PMX cartridge, respectively. The primary efficacy analyses will test the null hypothesis of *H*_0_ : *P*_1_ = *P*_0_ against the two-sided alternative hypothesis of *H*_*a*_ : *P*_1_ ≠ *P*_0_. The test of these hypotheses will be carried out through Fisher’s exact test [29]. A two sided 5% significance level will be assumed for the primary efficacy test. Similar analyses on the primary efficacy endpoint will also be conducted on the per protocol population.

In addition, a long-term follow-up of survival status at 90 days, 6 months and 12 months will be undertaken for the ITT population. This will entail a comparison of survival rates for ITT subjects at 90 days, 6 months and 12 months after assignment to either the treatment perfusion or the sham control perfusion group.

#### Safety and the data safety monitoring board

An external, independent DSMB was established. The DSMB acts in an advisory capacity to the Sponsor to monitor subject safety, including mortality, which is also the primary efficacy endpoint of the intervention. The DSMB comprises a group of individuals with a broad and varied base of pertinent expertise who will review the accumulating safety data on a quarterly basis. The DSMB consists of a microbiologist, a nephrologist, a critical care physician, a biostatistician, and an expert in clinical trial design. The meetings consist of an open session blinded report and include participation by the sponsor and their representatives from the clinical research organization trial operations team and a closed session of only DSMB members and one unblinded representative from the CRO. The closed session report of data tables and listings from the database is presented in a blinded manner. The DSMB has the right to ask for unblinding any portion of the closed session report if they deem it necessary. The DSMB will conduct an unblinded interim analysis of the safety data when half of the subjects have completed the first 28 days of the trial. The DSMB will also provide expert opinion on the clinical relevance of any safety findings, address any safety issues mentioned by the sponsor and, where applicable based on the safety data, advise the Sponsor as to the appropriateness of continuing the trial according to the protocol or modifying the protocol. The DSMB may terminate the trial if they deem it necessary for the protection of the safety of potential or current participants.

DSMB members are reimbursed for reasonable expenses related to attending meetings, such as travel costs, accommodations, and meals. Members are compensated for their time spent performing their responsibilities as members of the DSMB. No other payment or future consideration is provided. No formal relationship exists between the DSMB and any other committee involved in the EUPHRATES trial.

## Discussion

There are several unique features of the EUPHRATES trial compared to prior severe sepsis/septic shock studies. The trial does not include an assessment for features of the systemic inflammatory response syndrome as part of standard inclusion criteria. These criteria lack specificity and have been heavily criticized [30]. As an alternative, EUPHRATES targets patients with persistent shock and endotoxemia - aligning the purported mechanism of the treatment effect in hemodynamic improvement and the treatment of the presumed mediator of the disease process.

EUPHRATES is the first clinical trial in septic shock that we are aware of, designed to specifically use a biomarker as enrollment criteria for a therapy directed against that biomarker. This “theragnostic” approach has already proven successful in disease areas such as cancer and offers the opportunity to move beyond the constraints of the syndrome-based definition of sepsis to biologically targeted therapy [4,31]. However, this approach by definition will limit the application of this therapy to only a part of our existing patient population with septic shock and endotoxemia, making the need to search for both new markers and new therapies even more important for those patients who do not have endotoxemia. Nevertheless, the approach may be a better way to conduct sepsis trials using smaller numbers of patients with higher efficacy hurdles. In this trial, if one were to assume that all patients were to be enrolled based on meeting clinical criteria alone and that 50% of those patients enrolled were not likely to benefit from the treatment, a required efficacy hurdle of 7.5% would be needed or a trial of nearly 1,200 patients. The duration, cost and complexity of such a trial would likely be prohibitive.

Finally, EUPHRATES is the first trial of a hemoperfusion device to our knowledge that will be blinded. While blinding offers significant advantages in the overall strength of the study, the use of a “sham” perfusion event rather than true sham perfusion was chosen because sham perfusion was felt to be unethical and invasive [32]. Blinding a trial of this nature where patients are unstable and constantly interacting with numerous health-care providers is challenging and it is our intention to study the overall strength of the blinding procedures at the end of this trial so that lessons may be applied to future trials of this nature. This will be done by tracking all unblindings that occur, who was unblinded and how the unblinding occurred.

Thus, EUPHRATES is a pivotal trial using PMX hemoperfusion in patients with septic shock and confirmed endotoxemia. While the results of recent sepsis trials have been disappointing, there are several unique features of EUPHRATES, including its theragnostic design, that we hope are a step forward in the design of clinical trials for this underserved patient population.

## Trial status

The trial, at the time of submission of this manuscript, has enrolled approximately 275 subjects with all anticipated centers activated and trained. The second interim analysis was in the first quarter of 2014. Outcomes included a positive recommendation to continue the trial, sample size recalculation to a new total size of 650 patients, and addition of an exclusion criteria for multiple organ dysfunction score ≤9.

## Abbreviations

C4: Cooper University Hospital clinical coordinating center; CRF: case report form; DSMB: data safety monitoring board; EAA: endotoxin activity assay; EUPHRATES: Evaluating the Use of Polymyxin B Hemoperfusion in a Randomized controlled trial of Adults Treated for Endotoxemia and Septic shock; FDA: Food and Drug Administration; ITT: intention to treat; LPS: lipopolysaccharide; PMX: Polymyxin B; PMX-DHP: Polymyxin B direct hemoperfusion; SSC: Surviving Sepsis Campaign; TLR: Toll-like receptor.

## Competing interests

PMW and DF are employees of Spectral Diagnostics Inc. KK is an employee of Amarex, the clinical research organization conducting the trial. DJK has served as a consultant to Spectral Diagnostics Inc. The institutions of RPD and CAS have received research funding and consulting fees (steering committee for trial) from Spectral Diagnostics Inc.

## Authors’ contributions

DJK: conception and design, manuscript writing and final approval of the manuscript. DF: conception and design, manuscript writing and final approval of the manuscript. CAS: conception and design, data collection, manuscript editing and final approval of the manuscript. KK: statistical design, data analysis, manuscript writing and final approval of the manuscript. PMW: conception and design, critical revision of the manuscript, and final approval of the manuscript. RPD: conception and design, critical revision of the manuscript, and final approval of the manuscript. All authors’ agree to be accountable for all aspects of the work.
